# Immunopathogenesis of accelerated atherosclerosis in systemic lupus erythematosus: from innate and adaptive dysregulation to clinical implications

**DOI:** 10.3389/fimmu.2026.1766970

**Published:** 2026-03-04

**Authors:** Shirui Cao, Cheng Wang

**Affiliations:** 1 Department of Rheumatology, Union Hospital, Tongji Medical College, Huazhong University of Science and Technology, Wuhan, China; 2 Clinic Center of Human Gene Research, Union Hospital, Tongji Medical College, Huazhong University of Science and Technology, Wuhan, China; 3 Hubei Key Laboratory of Metabolic Abnormalities and Vascular Aging, Huazhong University of Science and Technology, Wuhan, China; 4 Hubei Clinical Research Center for Metabolic and Cardiovascular Disease, Huazhong University of Science and Technology, Wuhan, China

**Keywords:** atherosclerosis, autoimmunity, cytokines, immune mechanism, NETosis, systemic lupus erythematosus, type I interferon

## Abstract

Systemic lupus erythematosus (SLE) is a systemic autoimmune disease associated with significantly accelerated atherosclerosis (AS) and increased cardiovascular risk. This review elucidates the complex immunopathological mechanisms through which SLE promotes AS, involving both innate and adaptive immune dysregulation. Aberrant activation of the type I interferon signaling pathway and dysfunctional neutrophil/NETosis reciprocally amplify each other, forming a core upstream positive feedback loop. This loop accelerates atherosclerosis progression through multiple pathways, including driving endothelial dysfunction, promoting foam cell formation, and undermining plaque stability. Dysregulation of innate immune cells is prominent. Monocytes and macrophages exhibit altered polarization and impaired efferocytosis. Complement activation further exacerbates vascular injury. Within the adaptive immune system, T-cell subsets are imbalanced, promoting inflammation and AS progression. B cells and autoantibodies play dual roles. Although certain natural IgM antibodies may be protective, class-switched IgG autoantibodies often promote atherosclerosis. The role of B-cell activating factor (BAFF) and its inhibition in AS remains complex and context dependent. Animal models have been instrumental in dissecting these pathways, revealing interactions between lupus-like autoimmunity and atherogenic processes. Despite these advances, accurately assessing cardiovascular risk in SLE patients remains challenging, underscoring the need for SLE-specific risk prediction tools. Future directions should focus on identifying specific immune mechanisms, developing targeted immunomodulatory therapies, and establishing improved risk stratification strategies to enable early intervention and improve long-term outcomes for patients with SLE.

## 1 Introduction

Systemic lupus erythematosus (SLE) is a prevalent systemic autoimmune disorder characterized by the involvement of multiple systems (1). Cardiovascular involvement is especially prevalent in SLE patients (2). The pathogenesis of SLE and its associated cardiovascular damage involves complex interactions among multiple pathological mechanisms, such as dysregulated innate and adaptive immune activation, deposition of immune complexes, and sustained tissue inflammation (3). Together, these processes contribute to a range of adverse cardiovascular outcomes, including pericarditis, atherosclerotic events—such as stroke and myocardial infarction—and venous thromboembolism, which can be life-threatening in severe instances (4, 5). Among these, atherosclerosis, as a key initiating factor of many cardiovascular events, exhibits higher prevalence and increased susceptibility in SLE patients compared with the general population. However, the absence of SLE-specific risk assessment tools and biomarkers poses considerable challenges for the early detection of concomitant atherosclerosis, frequently resulting in delayed diagnosis. Therefore, elucidating the pathophysiological mechanisms through which SLE promotes atherosclerosis is essential for improving early diagnosis, enabling timely intervention, and guiding the development of more targeted diagnostic and therapeutic approaches (6).

### 1.1 Epidemiological evidence and risk stratification challenges

Atherosclerosis is recognized as a major cause of disability and mortality in patients with SLE (7, 8). Coronary artery disease accounts for up to 30% of deaths among SLE patients (9). The prevalence of atherosclerotic vascular events increases from 1.8% in early disease to more than 27% in longstanding SLE (10). Compared with the general population, patients with SLE have both a higher incidence of atherosclerosis and a greater likelihood of adverse cardiovascular outcomes. The risk of subclinical atherosclerosis progression in patients with SLE is four times greater than that in age- and sex-matched healthy controls (11, 12). This difference is more pronounced among young women of childbearing age. The relative risk of myocardial infarction in SLE patients aged 35–44 is 52.4 times greater than that of their peers (13). Recent Mendelian randomization analyses suggest a potential causal relationship between a genetic predisposition to SLE and an increased risk of atherosclerotic peripheral arterial disease (PAD) (14). A study in 1976 first reported a bimodal mortality pattern in patients with SLE, in which early deaths were due to active lupus, whereas later deaths were associated with a startlingly high incidence of myocardial infarction caused by atherosclerotic heart disease (15). As research into the mechanisms of atherosclerosis deepens, chronic arterial inflammation is recognized as a fundamental pathogenic mechanism, drawing increasing attention to the potential role of autoimmune diseases (16, 17). A comparison of 80 female SLE patients with 241 controls from the Multi-Ethnic Study of Atherosclerosis (MESA), after adjusting for age, race, education, income, diabetes, hypertension, dyslipidemia, HDL levels, smoking status, and BMI, revealed a significantly higher prevalence of coronary artery calcification in the SLE group (18). Although controlling traditional cardiovascular disease risk factors helps reduce the incidence of AS in patients with SLE, traditional Framingham risk factors cannot fully account for the accelerated atherosclerosis observed in patients with systemic lupus erythematosus (19). Statistical analyses have confirmed that SLE is an independent risk factor for atherosclerosis (20). In addition, the risk of cardiovascular disease (CVD) is significantly higher in patients with lupus nephritis (LN) than in those with SLE alone (7, 21).

General cardiovascular risk (CVR) assessment tools, such as the Framingham Risk Score (FRS) and the Systemic Coronary Risk Evaluation (SCORE), usually underestimate risk in patients with SLE and antiphospholipid syndrome (APS), particularly regarding stroke risk. The European League Against Rheumatism (EULAR) published its guidelines on cardiovascular risk management in rheumatic and musculoskeletal diseases, recommending that risk factor interventions be guided by assessments of both traditional cardiovascular risk factors and disease-specific risk factors in patients with SLE (22). However, the guidelines do not explicitly recommend any specific assessment tools.

Risk assessment tools for atherosclerosis in systemic lupus erythematosus (SLE) have evolved over time. They have moved from generic tools to modified versions. And now they are becoming SLE-specific tools (23). Researchers have developed two main strategies to improve risk assessment of generic tools. One strategy applies multiplicative adjustments to generic risk scores. Among the various tools, QRISK3, which incorporates SLE as a risk variable, and the modified Framingham Risk Score (mFRS) outperform the original FRS and SCORE in predicting atherosclerosis progression (24, 25). Both have been validated in cohorts to identify a greater proportion of high-risk patients (26). However, these modified tools have significant limitations. They do not fully account for SLE-specific risk factors. This leads to substantial underestimation of cardiovascular risk. Their clinical utility remains limited when used alone (22, 23).

Therefore, researchers have developed SLE-specific prediction tools. SLECRISK represents one such specialized tool. It incorporates multiple disease-specific parameters. SLECRISK integrates multiple disease-specific parameters, achieving a sensitivity of 74% for predicting moderate-to-high risk major adverse cardiovascular events (MACE)—nearly double that of the ACC/AHA model (38%) (5). However, direct comparisons between SLECRISK and modified generic models are still needed.

The application of high-throughput sequencing provides new possibilities for more accurately calculating the potential risk of AS in patients with SLE. Researchers from Peking Union Medical College Hospital conducted RNA sequencing on 67 SLE patients and developed an atherosclerosis risk prediction model that includes age, hyperlipidemia, and Keratin 10 (KRT10) (27). This model achieved optimal discriminative performance using just three straightforward variables. KRT10 gene expression serves as a novel biomarker with promising application potential. However, urgent resolution is required to address the limitations of small sample size and lack of external validation. Recent studies have shown that lipoprotein subclasses in serum metabolites, such as free cholesterol in VLDL particles and HDL2, also represent potential biomarkers that may be used to predict subclinical atherosclerosis in patients with SLE in the future (28, 29).

In summary, it is recommended that clinical practice prioritize the use of modified risk assessment tools that integrate disease-specific factors, such as QRISK3 or mFRS, for initial screening. At the same time, it is essential to actively monitor and manage SLE-specific risk factors, such as cumulative glucocorticoid dose and antiphospholipid antibody status, in order to achieve more precise cardiovascular risk stratification and intervention (23). Although emerging measurement tools show positive potential, they require further external validation (30). The development of tools that precisely quantify atherosclerotic potential and plaque burden in SLE patients will greatly assist rheumatologists in cardiovascular risk management.

### 1.2 Preclinical models: dissecting the SLE-atherosclerosis interplay

Researchers have established various animal models to study the accelerating effect of SLE on AS. There are three main types, which are spontaneous, combined, and purely induced models (**Table 1**). Spontaneous models are mostly inbred strains, such as NZB/W F1, MRL/lpr, and BXSB/Yaa. These models can naturally develop both SLE and atherosclerosis, mimicking the complex immune and metabolic disorders in patients with SLE (31).

**Table 1:** Lupus-like manifestations and atherosclerosis lesions associated with different SLE-AS mouse models.

Model		Sex bias	Lupus-like manifestations	Atherosclerosis lesions	Reference
Inbred strains	MRL/MpJ-Faslpr (MRL/lpr)	both(female slightly earlier)	Autoantibody production, massive lymphadenopathy, splenomegaly, LN, skin rash, overt arthritis and cutaneous vasculitis, NPSLE at an early stage of disease	Vascular and perivascular leukocytic infiltration, increased IMT of the aorta, lipid accumulation in coronary arteries, presence of immune complexes in vessel walls, endothelial dysfunction, higher incidence of MI	(88, 173–177)
	(NZB×NZW) F1	female	Predominantly anti-dsDNA antibodies, anti-Sm antibodies are rare, target organs are mainly kidneys	Increased heart weight, hypertrophy of the LV and septum, increased IMT of the aorta, endothelial dysfunction, increased cardiomyocyte apoptosis	(178–181)
	BXSB/MpJ-Yaa (BXSB/Yaa)	Male	Modest lymphoproliferation; prominent monocytosis, hyper-gammaglobulinaemia, LN	Increased risk of myocardial infarction, increased atherosclerosis susceptibility	(31, 182)
	NZM2410	female	Similar to (NZB×NZW) F1 but faster nephritis	Enhanced atherosclerosis in LDLr^-^/^-^ or ApoE^-^/^-^ mice	(31, 183, 184)
Induced models	Pristane induced	female	Autoantibody production, lymphadenopathy, splenomegaly, LN, valuable for environment-triggered NPSLE	Increased IMT, lipid plaques, cholesterol crystals, calcification	(185)
	Pristane + BCG Injection	female	Autoantibody production, lymphadenopathy, splenomegaly, LN, skin rash, arthritis and cutaneous vasculitis, hyperactivation of T and B cells	perivascular leukocytic and macrophage infiltration, lipid accumulation in coronary arteries	(186)
Hybrid model	ApoE^-^/^-^Fas^-^/^-^	both	Autoantibody production, lymphadenopathy, splenomegaly, LN, skin rash, arthritis and cutaneous vasculitis	Promote plaque progression, activation of endothelial cell adhesion molecules, promoting foam cell formation, increased infiltration of inflammatory cells within the plaque	(187)
	gld. ApoE^-^/^-^	both	Amplified autoimmune abnormalities, earlier onset of disease, cytokine Imbalance, autoantibody production, lymphadenopathy, splenomegaly	Lipid metabolism disorde, endothelial dysfunction, lipid accumulation in coronary arteries	(188)
	ApoE^-^/^-^Nba2.Yaa (Yaa × ApoE^-^/^-^)	male	Autoantibody production, worsened LN with male predominance	Characteristics of atherosclerotic plaque destabilization associated with the rise in pro-atherogenic autoantibodies	(189)
Genetically sensitized and chemically triggered model	LDLr^-^/^-^ + pristane	female	Autoantibody production, lymphadenopathy, splenomegaly, LN,	Endothelial dysfunction, lipid accumulation in coronary arteries	(37)
	ApoE^-^/^-^ + pristane	female	Autoantibody production, lymphadenopathy, splenomegaly, LN	Plaque burden significantly increased and stability decreased	(190)

This table is based on a comprehensive literature search of the PubMed database, focusing on original research articles and reviews published in the last two decades. The search utilized a combination of keywords and Medical Subject Headings (MeSH) terms, including ("systemic lupus erythematosus" OR SLE) AND (atherosclerosis OR "arterial lesions") AND (mouse OR murine) AND (model), to identify studies investigating the interplay between lupus pathogenesis and atherosclerotic cardiovascular disease in experimental murine systems. LN, Lupus Nephritis; IMT, Intima media thickness; MI, Myocardial infarction; BCG, Bacillus Calmette-Guérin; LV, Left ventricle; HFD, High fat diet.

Another commonly used model in the study is the combination type, which can be further divided into the hybrid model and the genetically sensitized and chemically triggered model. The hybrid model involves crossbreeding mouse models susceptible to SLE and AS, such as the spontaneous models gld.apoE^-/-^ and ApoE^-/-^Fas^-/-^C57BL/6 mice (32). These models often exhibit severe autoimmune symptoms and rapid atherosclerosis formation (33). Induction can be achieved by injecting drugs or by transplanting bone marrow from lupus-susceptible mice into AS-susceptible mice (34), thereby causing lupus-like disease in AS-susceptible mice. Most people believe that SLE is a complex polygenic disease. Therefore, researchers have developed an animal model of radiation chimeras by irradiating atherosclerosis-prone low-density lipoprotein receptor-deficient (LDLr^−/−^) mice with lethal doses and combining them with B6.Sle1.2.3 triple congenic animals, reporting that only mice containing all three congenic genes can express the full range of SLE disease manifestations (35). Another type of model commonly uses LDLr^-/-^ or APOE^-/-^ mice with spontaneous atherosclerosis, which are intraperitoneally injected with pristane to induce SLE (36, 37). This model can also be combined with a high-fat diet to accelerate the formation of atherosclerosis. Compared with other models, this model has a shorter induction time, but it often fails to exhibit the full phenotype of SLE. Moreover, there are individual differences in drug susceptibility among mice.

Recent studies have shown that SLE-AS models can also be established purely by induction. Specifically, intraperitoneal injection of pristane into C57BL/6J mice followed by intradermal injection of Bacillus Calmette–Guérin (BCG) one month later resulted in the successful establishment of a mouse model of SLE with atherosclerosis (AS). Combined injection of BCG significantly exacerbates vascular inflammation and increases aortic intima-media thickness (IMT), as well as perivascular lymphocyte and macrophage infiltration and lipid deposition (28). This model provides an efficient and cost-effective experimental platform to study the development mechanisms of atherosclerosis in the context of SLE.

Although murine models remain indispensable for mechanistic exploration, significant translational barriers limit their clinical fidelity in SLE-associated atherosclerosis (SLE-AS). These limitations span metabolic, sexual dimorphic, and temporal dimensions.

Species-specific lipid metabolism is a primary constraint. Human SLE patients exhibit high-density lipoprotein (HDL) dysfunction and elevated low-density lipoprotein (LDL). Murine models utilize distinct liver X receptor (LXR)-ATP-binding cassette transporter (ABCA1/G1) pathways (38). They lack estrogen-driven modulation of HDL particle size (39). Consequently, these models fail to replicate the protective lipid metabolic phenomena specific to female SLE patients (40).

Sex bias simulation remains inadequate (41). The vast majority of SLE-AS studies use only female mice. Although male patients have lower disease incidence, they demonstrate worse outcomes for lupus nephritis and cardiovascular prognosis. Murine models display divergent patterns. The frequency of hormonal fluctuations also diverges from human patterns (42, 43).

Chronicity and heterogeneity are poorly replicated. Human SLE-AS evolves over decades. Persistent inflammation, intermittent flares, and immunosuppressant exposure drive vascular remodeling. Induced models produce acute interferon responses (44). They fail to capture chronic low-density neutrophils (LDNs) expansion. Spontaneous models carry monogenic defects. These cannot reflect human polygenic architecture. Renal and vascular pathology differ anatomically and molecularly from human disease (45).

## 2 Central immunopathological mechanisms in SLE-accelerated atherosclerosis

### 2.1 The IFN-I/NETosis positive feedback loop as the primary upstream driver

**Figure 1:**
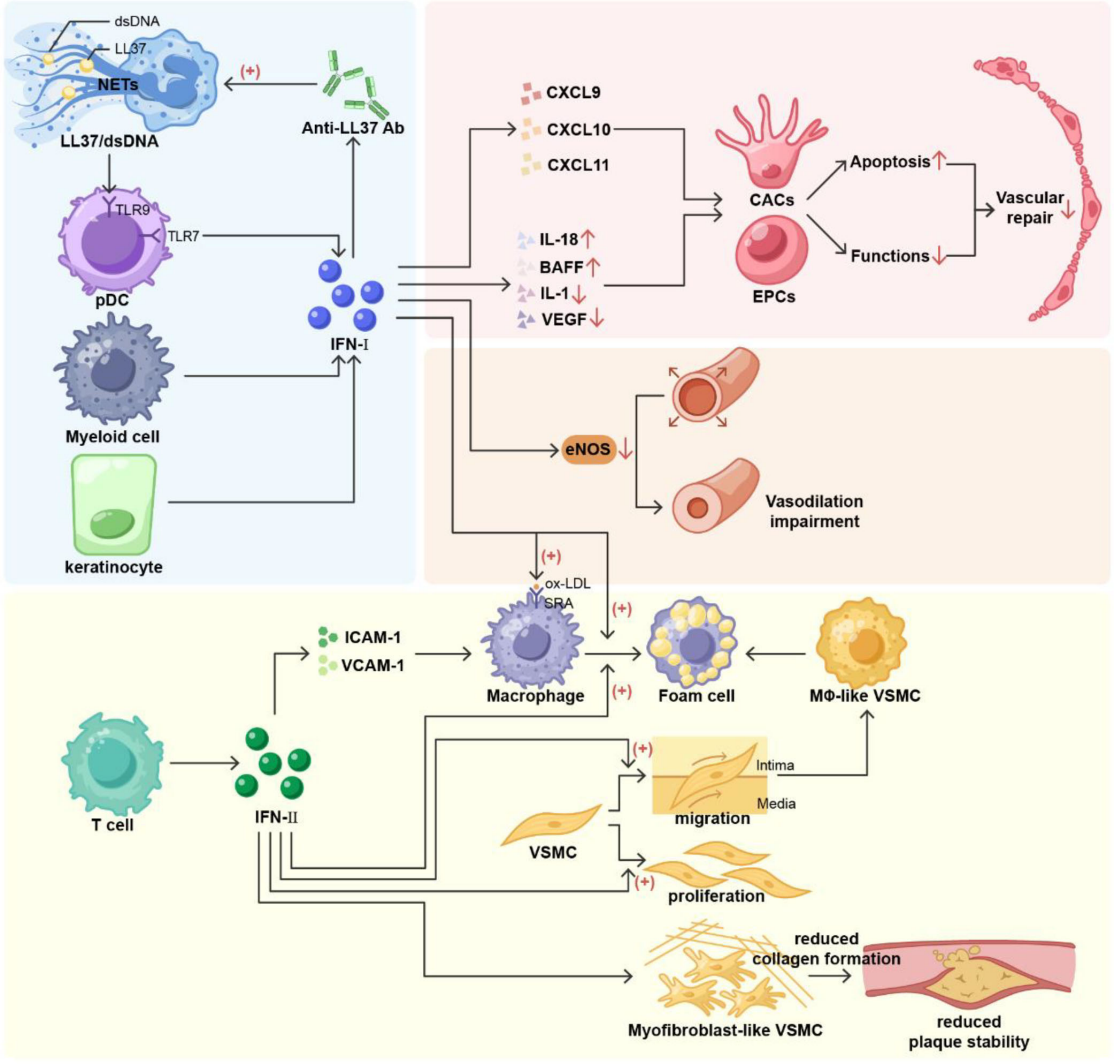
The core role of interferon in promoting atherosclerosis in SLE. In systemic lupus erythematosus (SLE), type I interferon (IFN-I) likely originates from myeloid cells and dendritic cells. Keratinocytes represent a newly identified source. IFN-I promotes anti-LL37 antibody production, leading to increased LL37/dsDNA in neutrophil extracellular traps (NETs). LL-37 facilitates its internalization within plasmacytoid dendritic cells (pDCs) and induces Toll-like receptor 9 (TLR9) activation, forming a positive feedback pathway for IFN-I generation. IFN-I actively induces ELR-negative CXC chemokines, enhances IL-18 and B-cell activating factor (BAFF) effects, and suppresses IL-1 and vascular endothelial growth factor (VEGF) expression. Collectively, these actions precipitate increased apoptosis and functional impairment in endothelial progenitor cells (EPCs) and circulating myeloid angiogenic cells (CACs), alongside impaired vascular repair. IFN-I increases endothelial nitric oxide synthase (eNOS) degradation, leading to diminished vasodilatory capacity. Within macrophages, the IFN-I signaling pathway upregulates expression of the scavenger receptor A (SRA) on class A macrophages, which binds oxidized low-density lipoprotein, resulting in foam cell formation. Type II Interferon(IFN-II)is primarily secreted by T cells. It promotes vascular cell adhesion molecule-1 (VCAM-1) and intercellular adhesion molecule-1 (ICAM-1), enhancing monocyte adhesion and facilitating foam cell accumulation. IFN-II stimulates vascular smooth muscle cell (VSMC) proliferation and migration into the arterial intima, mediating VSMC phenotypic conversion to form smooth muscle-derived foam cells. IFN-II reduces collagen fiber production in myofibroblast-like VSMC fibrous caps, destabilizing plaque structure. IFN-I, type I interferon; Ab, antibody; dsDNA, double stranded DNA; NETs, neutrophil extracellular traps; pDCs, plasmacytoid dendritic cells; TLR9, Toll-like receptor 9; TLR7, Toll-like receptor 7; EPCs, endothelial progenitor cells; CACs, circulating myeloid angiogenic cells; BAFF B-cell activating factor; VEGF, vascular endothelial growth factor; eNOS, endothelial nitric oxide synthase; SRA, scavenger receptor A; IFN-II, Type II Interferon; VCAM-1, vascular cell adhesion molecule-1; ICAM-1, intercellular adhesion molecule-1; VSMC, vascular smooth muscle cell.

SLE accelerates AS through a complex immune network. The abnormal activation of the type I interferon (IFN-I) signaling pathway, coupled with dysregulated neutrophil/NETosis, represents the core upstream driver of the disease. Interferon plays a central role in both the pathogenesis of systemic lupus erythematosus and the development of atherosclerosis (**Figure 1**). In SLE, overactivation of the IFN-I signaling pathway is particularly important. A correlation exists between the IFN-I signature and the SLE Disease Activity Index (SLEDAI), which is used to measure the severity of the disease (46). Single-cell transcriptomic analysis of bone marrow cells from patients with SLE revealed that their HSPCs exhibit IFN-related features early on, with alterations in the cell cycle and DNA repair mechanisms, and these changes are passed on to their progeny cells (47). Interferon-inducible reprogramming in HSPCs potentially represents an initiating event in this disease, but more studies are needed. More than 50% of SLE patients have persistent IFN-I signaling activation, which is significantly associated with early-onset cardiovascular events (48). Moreover, during disease progression, the increase in IFN-I levels in the circulation of patients with SLE has been identified as a significant factor contributing to the worsening of atherosclerosis (49, 50).

IFN-I triggers neutrophils to translocate LL-37 to the cell surface. After binding to anti-LL-37 antibodies, the LL37-dsDNA complex is released with NETs, and LL-37 promotes their internalization into pDCs and induces TLR9 activation (51). This positive feedback loop between NETs and IFN-I produced by pDCs represents a pathogenic factor exacerbating atherosclerosis in patients with SLE (52).

Myeloid cells sense cytoplasmic DNA through the cGAS-STING pathway, leading to the secretion of IFN-β and promoting the release of inflammatory cytokines (53). In another study, increased IFN-I signaling pathway activity was found in TRIM21-deficient mice that exacerbated the inflammatory response induced by ultraviolet B (UVB), revealing a potential link between photosensitivity in SLE and systemic IFN-I inflammation (54).

IFN-I drives multiple pathways that culminate in endothelial dysfunction and impaired vascular repair, key processes in atherosclerosis (55). The centrality of IFN-I in these pathogenic processes is confirmed by interventional and genetic studies. Acute interferon exposure recapitulates the impairment of endothelial vasodilation and EPC function, whereas apoE-/-IFNAR-/- mice lacking the type I interferon receptor exhibit significant improvements, including enhanced endothelium-dependent vasodilation and improved EPC number and function (56).IFN-I promotes foam cell formation by enhancing the uptake of modified lipoproteins by macrophages (57). IFN-I enhances antigen-presenting capacity, promotes dendritic cell maturation, stimulates B-cell differentiation into autoantibody-producing cells, and induces CD8^+^ T cells to differentiate into cytotoxic effector cells, thereby leading to a persistent state of autoimmunity and indirectly promoting vascular inflammation.

In addition to IFN-I, IFN-II is involved in the pathogenesis of SLE (58). Studies have revealed significant differences in interferon-type levels between the clinical and transcriptional phenotypes in patients with SLE, with an additive effect of interferons in severe SLE. In addition, some systemic symptoms are associated with the co-elevation of IFN-I, IFN-II, and IFN-III (59). IFN-II is mainly secreted by T cells and activates various cellular pathways, primarily the Janus kinase (JAK)/signal transducer and activator of transcription (STAT) pathway (60). IFN-γ impairs endothelial glucose metabolism by altering tryptophan catabolism, disrupting HIF1, and depleting NAD+, leading to a metabolic shift towards increased fatty acid oxidation (61). Moreover, IFN-γ enhances the acquisition of large amounts of cholesteryl esters by macrophages through the phagocytosis of apoptotic cells (62). In experiments involving macrophage polarization analysis of RAW264.7 cells, LPS and IFN-γ induced macrophages to exhibit an M1 phenotype (63), thereby promoting the formation of foam cells. IFN-γ promotes the migration and infiltration of monocytes and T lymphocytes beneath the endothelium by inducing the expression of adhesion molecules such as ICAM-1 and VCAM-1, thereby initiating an inflammatory response (64). IFN-γ upregulates mini-Trp RS-mediated phenotypic switching of vascular smooth muscle cells (VSMCs), which is the basis for the formation of smooth muscle-derived foam cells (65). IFN-γ also inhibits collagen production by VSMCs, an effect that may weaken plaque stability (66).

Neutrophil hyperactivation and neutrophil extracellular trap (NET) formation play multiple roles in SLE vascular damage (67). Neutrophils in patients with SLE exhibit impaired phagocytic clearance, increased apoptosis, and abnormal oxidative metabolism (68). A heterogeneous subpopulation of low-density granulocytes (LDGs) composed of mature CD10^hi^ and immature CD10^lo^ neutrophils is present in the peripheral blood of patients, and CD10^hi^ neutrophils are associated with SLE disease activity. LDGs have strong proinflammatory properties (69) and produce high levels of IFN-I and other inflammatory cytokines, such as TNF and IFN-γ (70). NET formation is accompanied by neutrophil death, termed NETosis, which is closely related to reactive oxygen species (ROS) generated by NADPH oxidase and mitochondria. Increased mROS production in LDGs promotes the spontaneous formation of NETs rich in oxidized mitochondrial DNA. Compared with native DNA, oxidized DNA is more immunogenic and has a stronger ability to induce IFN-α production by pDCs (71). ROS produced by these cells can also increase vascular permeability and convert LDL into low-density lipoprotein (ox-LDL), which accumulates beneath the endothelium (72). Ox-LDL is particularly important in atherosclerosis and induces inflammation through scavenger receptors and Toll-like receptors (TLRs) (73). Compared with NETs from healthy controls and lupus neutrophils, lupus LDG NETs exhibit various posttranslational modifications, affecting their physiological functions (72). Specifically, in NETs derived from lupus LDGs, the NET-associated microRNA let-7b is expressed at high levels. This small RNA is internalized by ECs, acts as a TLR-7 agonist, and induces the expression of ISGs (74). LDGs show differential phosphorylation of proteins related to cytoskeletal organization, resulting in reduced cell deformability and a rougher membrane surface. LDGs that remain in the microvascular network cause damage (75). Neutrophil elastase in NETs degrades collagen in the fibrous cap, weakening plaque stability. Studies have shown that polyubiquitinated protein expression is reduced in NETs from patients with SLE, with myeloperoxidase (MPO) present in its ubiquitinated form. SLE patients also produce antibodies against ubiquitinated MPO. *In vitro* experiments have shown that both NETs and ubiquitin can activate macrophage calcium influx and promote TNF-α and interleukin-10 production in macrophages from patients with SLE. This finding suggests that abnormalities in ubiquitinated proteins in NETs may contribute to the development of inflammation in patients with SLE through the activation of adaptive immune responses and macrophage inflammatory responses (76). Moreover, NETs act as a scaffold to capture platelets and red blood cells, promoting arterial thrombosis (77).

In addition to NETs, macrophages can also release METs in a similar manner. Compared with those from healthy individuals, monocytes from active SLE patients are more prone to apoptosis and MET formation (78). However, its composition is not entirely the same as that of NETs, but it may promote AS in a similar manner.

### 2.2 Contributions of other pro-inflammatory cytokines

Tumor necrosis factor alpha (TNF-α) has recently been identified as a major regulator of inflammatory responses and a pleiotropic cytokine that acts on many types of cells (79). In young, clinically active SLE patients, TNF−α and hsCRP were positively correlated with pulse wave velocity, an early marker of arterial stiffness and vascular damage. This connects systemic inflammatory load to functional vascular impairment in human SLE levels (80, 81). From a genetic perspective, multiple studies have shown an association between TNF-α gene polymorphisms and susceptibility to systemic lupus erythematosus (82). Experiments have shown that the single-cell IFN-I response pattern in plasmacytoid dendritic cells (pDCs) of patients with SLE is similar to that of healthy individuals. However, the TNF-α system is significantly dysregulated, with enhanced IFN-β priming of TNF-α secretion and disruption of the pathological inhibitory relationship between IFN-I and TNF-α (83). The pathological crosstalk between IFN-I and TNF-α suggests that targeting a single cytokine may lead to compensatory dysregulation of another pathway. This mechanism has received indirect support from clinical observations. In some patients treated with anti-TNF-α drugs for rheumatoid arthritis, cases of induced antinuclear antibodies or extremely rare lupus-like syndromes have been reported, suggesting potential risks associated with disrupting the IFN-I/TNF-α balance (84).

IL-6 is a cytokine downstream of the IL-1 signaling cascade and is involved in both the innate and adaptive immune systems. Examination of serum from patients with SLE revealed increased IL-6 and MCP-1 concentrations, with IL-6 being correlated with the atherosclerotic burden in patients with SLE (85). IL-6 and CRP are well-validated inflammatory predictors of cardiovascular (CV) risk, with IL-6 occupying a more causal, upstream role (86). Animal lupus models and non-SLE autoimmune vascular disease support the concept that IL-6 signaling promotes vascular inflammation and dysfunction, but dedicated trials are still needed to determine whether IL-6–targeted therapies meaningfully reduce cardiovascular events in SLE patients (87, 88).

IL-17 has been implicated in the pathogenesis of SLE and is considered a novel therapeutic target (89). Its role extends to cardiovascular disease, where it is thought to bridge innate and adaptive immunity (90). Substantial preclinical evidence supports a pro-atherogenic role for IL-17. Elevated IL-17 levels are believed to promote atherosclerotic plaque formation and instability. In apolipoprotein E-deficient (apoE−/−) mice, functional blockade of IL-17 reduces both plaque burden and vulnerability, underscoring its critical role in disease progression (91). The mechanisms by which IL-17A drives atherosclerosis include enhancing immune cell infiltration and local inflammation, as well as promoting endothelial dysfunction and vascular apoptosis. Furthermore, the IL-17 family member IL-17D was recently shown to accelerate atherosclerosis by promoting endothelial ferroptosis via the CD93/miR-181a-5p/SLC7A11 signaling pathway (92).

Based on the aforementioned clinical and preclinical evidence, the progression of SLE-associated atherosclerosis exhibits distinct phase-specific cytokine characteristics. In the early phase, IFN-I plays a pivotal role as the key initiating factor (93). TNF-α also synergizes with IFN-I during this period to exacerbate initial injury by promoting endothelial oxidative stress and adhesion molecule expression (94). During progression, additional cytokine networks are recruited and amplified. TNF-α and IL-6 emerge as primary drivers (95, 96).In the late stage, the critical phase leading to plaque rupture and clinical events such as myocardial infarction, TNF-α and IL-17 are considered key destabilizing factors (97). They collectively contribute to plaque vulnerability by inhibiting collagen synthesis in vascular smooth muscle cells, promoting apoptosis, weakening the fibrous cap, and enhancing the activity of pro-inflammatory enzymes.

## 3 Innate immune dysregulation and vascular injury

### 3.1 Monocytes and macrophages: recruitment, polarization, and dysfunctional clearance

**Figure 2:**
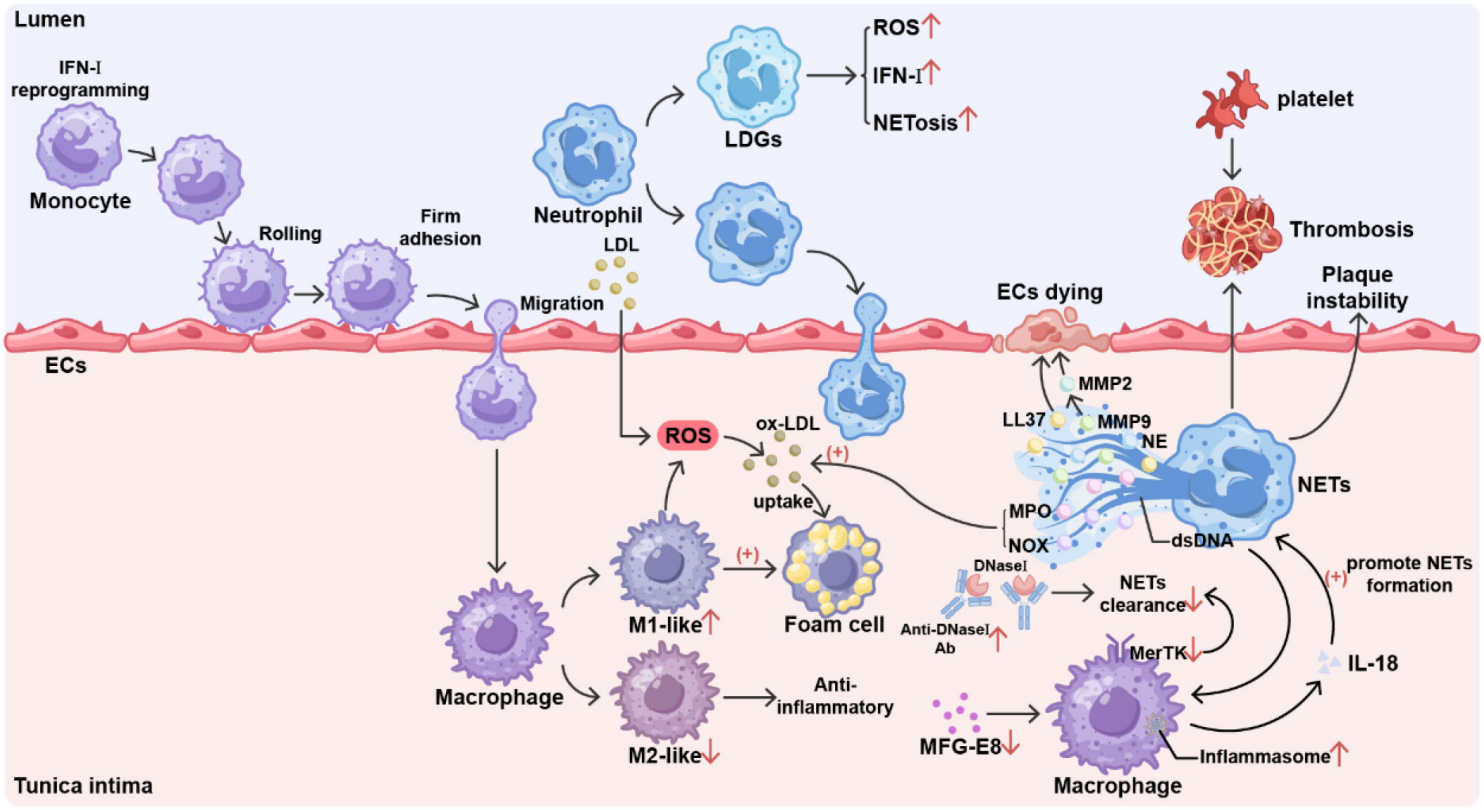
The primary role of the innate immune system in promoting atherosclerosis in SLE. In systemic lupus erythematosus (SLE), type I interferon (IFN-I) reprogrammed monocytes enter the arterial intima via rolling, adhesion, crawling, and transmigration, where they differentiate into macrophages and initiate lesion formation. SLE disrupts the developmental process of monocyte-macrophage cells, promoting M1 polarization and accelerating the transition to foam cells. This process is accompanied by altered intracellular metabolism, resulting in excessive reactive oxygen species (ROS) production. During atherosclerosis, M1 cells accelerate disease progression, whereas M2 cells inhibit it. ROS, along with the myeloperoxidase (MPO) and NADPH oxidases (NOX) in neutrophil extracellular traps (NETs), convert low-density lipoprotein (LDL) into oxidized low-density lipoprotein (ox-LDL). Ox-LDL uptake by macrophages, these cells transform into foam cells. Low-density granular cells (LDGs) are present in neutrophils from SLE patients. Compared to normal neutrophils, LDGs produce higher levels of ROS and IFN-I, and are exhibit increased susceptibility to NET formation. Neutrophils enter the tunica intima in a similar way to monocytes. SLE leads to increased NETs formation. The matrix metalloproteinase-9 (MMP-9) contained in NETs activates matrix metalloproteinase-2 (MMP-2) and the highly immunostimulatory LL-37 antimicrobial peptide, causing endothelial cell damage. NETs act as scaffolds to trap platelets and red blood cells, promoting intravascular thrombosis. Neutrophil elastase (NE) within NETs degrades collagen in the fibrous cap, which compromises plaque stability. The formation of NETs leads to increased inflammasome activation in adjacent macrophages, resulting in the release of inflammatory cytokines, such as IL-18. These cytokines further stimulate NET formation. Patients with SLE demonstrate a diminished capacity to clear NETs. One contributing factor is the presence of antibodies against DNase1, an endonuclease essential for NET degradation. Another factor is impaired phagocytic function in SLE macrophages. Downregulation of MerTK expression on SLE macrophage surfaces, coupled with deficiencies in chemotactic molecules such as MFG-E8, results in diminished clearance capacity. IFN-I, type I interferon; ROS, reactive oxygen species; MPO, myeloperoxidase; NOX, NADPH oxidases; NETs, neutrophil extracellular traps; LDL, low-density lipoprotein; ox-LDL, oxidized low-density lipoprotein; MMP-9, matrix metalloproteinase-9; MMP-2, matrix metalloproteinase-2; NE, neutrophil elastase; Ab, antibody.

The innate immune system promotes the progression of atherosclerosis via multiple underlying mechanisms in patients with SLE (**Figure 2**). The recruitment of blood monocytes is a key step in the development of atherosclerosis (98). In patients with SLE, the expression of adhesion molecules on endothelial cells, such as intercellular adhesion molecule-1 (ICAM-1) and vascular cell adhesion molecule-1 (VCAM-1), is increased (99). This increased expression promotes the adhesion and migration of monocytes to the endothelium and is weakly associated with disease activity. SLE affects the development of mononuclear macrophages, resulting in abnormal numbers, unique activation states, and functional impairments (47, 100). In SLE patients, the proportion of classical monocytes (cM) is significantly increased (101). During the active phase of SLE, macrophages tend to polarize towards the M1 phenotype, and nonclassical monocytes (ncM) also exhibit characteristics of M1 polarization (102), which can lead to increased plaque size and instability. Macrophage polarization is accompanied by changes in intracellular metabolism.

Another significant characteristic of macrophages in SLE is their impaired phagocytic capacity, which hinders the effective clearance of apoptotic and necrotic cells. Apoptotic cells that are not promptly cleared undergo secondary necrosis, releasing proinflammatory mediators and autoantigens that contribute to the formation of unstable plaques. In SLE patients, noncalcified plaques account for more than 50% of the total plaque burden, and these plaques are more prone to rupture, triggering acute cardiovascular events (103, 104).

The lipid metabolism capacity of macrophages is also altered in patients with SLE. Recently, bioinformatics approaches combining machine learning algorithms with single-cell sequencing analysis have identified core genes that are shared between SLE and MetS, both of which are potentially associated with mononuclear-macrophages (105, 106).

In SLE patients, the transformation of macrophages into foam cells is enhanced through multiple pathways (107). Increased ROS levels in patients with SLE result in persistent oxidative damage in the chronic inflammatory state and generates piHDL and high levels of oxidized ox-LDL. Unlike HDL, which typically has anti-atherosclerotic effects, piHDL promotes inflammation and is associated with carotid plaque progression and intima-media thickness (IMT) in patients with SLE (108). Oxidation of HDL also reduces its cholesterol efflux capacity in SLE (109).

### 3.2 Complement activation: a link between autoimmunity and vascular injury

In the pathogenesis of systemic lupus erythematosus (SLE), abnormal activation of the classical complement pathway is among the key links and is triggered mainly by immune complexes. This activation pathway mediates the chemotaxis and aggregation of neutrophils and monocytes through the generation of anaphylatoxins C3a and C5a and causes direct cell damage through the formation of membrane attack complexes (MACs) that perforate cell membranes. This cascade of reactions is not only considered an important pathological mechanism for tissue damage in SLE but also may promote the occurrence of chronic vascular lesions. A cross-sectional analysis revealed that platelet C4d (PC4d) is an independent risk factor for vascular events in patients with SLE and synergistically increases the risk of vascular lesions when it coexists with a lupus anticoagulant (LA) (110). Although the complement system is crucial for immune activity and tissue clearance, overactivation of this system may promote the development of cardiovascular disease. The complement system is the bridge connecting chronic inflammation within the plaque and acute thrombotic events, driving the key molecular mechanisms of plaque erosion (111). However, the exact role of complement in the development of atherosclerosis in SLE patients still needs further investigation.

## 4 Adaptive immunity in SLE-associated atherosclerosis

### 4.1 T cell imbalances and tertiary lymphoid structures

**Figure 3:**
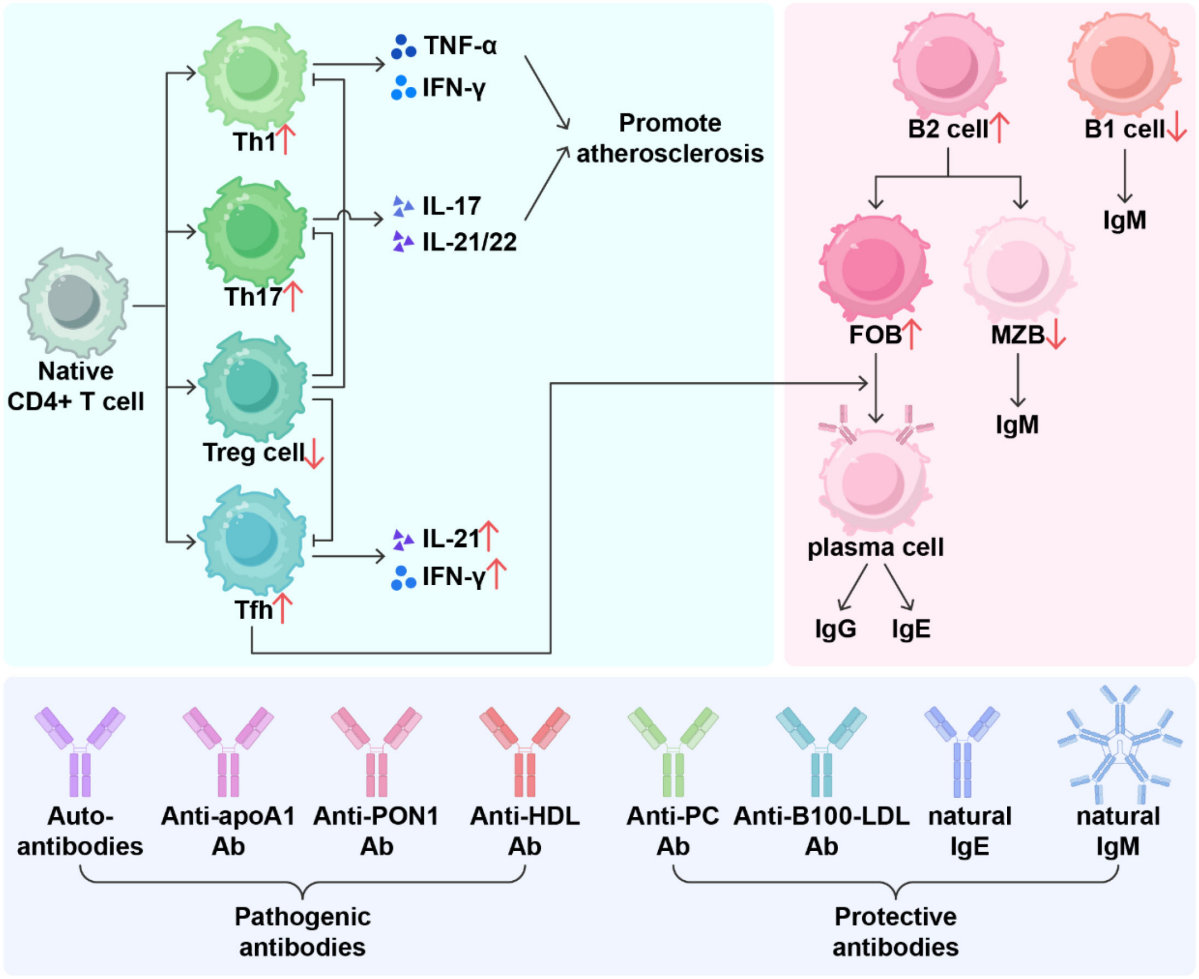
The multiple roles of adaptive immunity in promoting atherosclerosis in SLE. Systemic lupus erythematosus (SLE) leads to abnormal differentiation of T cell. Reduced regulatory T (Treg) cell function contributes to expansion of T helper 1 (Th1), T helper 17 (Th17) cells and T follicular helper (Tfh) cells. Th1 cells secrete potent pro-inflammatory factors, including IFN-γ and TNF-α. Th17 cells secrete IL-17 and IL-21/22, and Tfh cells secrete IL-21 and IFN-γ. All of these cytokines promote the development of atherosclerosis. B cells are central to the pathology of SLE. In SLE, B1 cells with anti-atherosclerotic effects are reduced, while B2 cells are increased. Marginal zone B (MZB) cells exert anti-atherosclerotic effects by regulating Tfh cells and secreting IgM, whereas follicular B (FOB) cells are considered pro-atherosclerotic. In SLE patients, MZB cells are reduced while FOB cells are increased. Under the influence of Tfh cells, FOB cells differentiate into plasma cells that produce IgG and IgE. B-cells produce pathogenic antibodies, such as auto-antibodies, anti-apoA1, anti-HDL and anti-PON1, which promote atherosclerosis. Meanwhile, protective antibodies, including natural IgG and IgE antibodies, anti-PC and anti-B100-LDL, prevent atherosclerosis. Th1, T helper 1; Th17, T helper 17; Treg, regulatory T; Tfh, T follicular helper; MZB, Marginal zone B; FOB, follicular B; Ab, antibody; apoA1, apolipoprotein A-I; HDL, high density lipoprotein; PON1, paraoxonase 1; PC, pyruvate carboxylase; B100, Apolipoprotein B100; LDL, Low-density lipoprotein.

Dysregulation of adaptive immunity plays a crucial role in the pathogenesis of lupus, but its contribution to atherosclerosis in patients with SLE remains underexplored (**Figure 3**). Lymphocytes were once thought to adhere to adhesion factors and enter the intima under the influence of chemokines induced by IFN-γ, similar to macrophages. However, subsequent studies have revealed that in early and nonprogressive atherosclerotic lesions, lymphocyte infiltration is sparse and concentrated mainly in the adventitia and media. As lesions progress, these cells form lymphoid-like structures in the adventitia, containing B cells and plasma cells, suggesting the presence of local immune reactions within human atherosclerotic plaques (112). These structures were later identified as tertiary lymphoid organs (TLOs). TLOs allow circulating T cells and B cells to interact with antigen-presenting cells (APCs) within their tightly structured environment (113). Using single-cell RNA sequencing, researchers have reported the involvement of TLOs in various disease models involving vascular remodeling (114). Although TLOs are more frequently mentioned in the context of lupus nephritis (LN) caused by SLE (115, 116), their structural specificity and role in atherosclerosis remain under investigated.

In SLE, the imbalance and dysfunction of T-cell subsets are the main mechanisms through which they contribute to cardiovascular disease. Cytokines and metabolic changes in the local microenvironment of cells in SLE patients lead to abnormal differentiation of Th0 (117, 118). Among CD4+ T cells, the ratio of Th1 and Th2 subsets is imbalanced, with Th1 cells being dominant during active SLE (119). The ratios of Th17 and Treg subsets are also imbalanced, with Th17 cells expanding and secreting IL-17 and IL-21/22, which promote endothelial dysfunction and neutrophil/monocyte recruitment and stimulate vascular smooth muscle cell migration and proliferation. Tregs have a protective effect against atherosclerosis. Dysfunction of Tregs, characterized by reduced numbers or decreased suppressive capacity, leads to uncontrolled autoimmune responses and failure to inhibit atherosclerosis (AS) inflammation (17).

T follicular helper (Tfh) cells are a CD4+ T-cell subset that promotes germinal center (GC) formation, antibody affinity maturation, and memory B-cell generation (120, 121). In SLE patients, the percentages of cTfh1 and cTfh2 cells are comparable to those in healthy controls (HCs), whereas the percentage of cTfh17 cells is greater than that in HCs (122). In SLE patients, T-cell senescence markers, including carotid intima-media thickness (cIMT), flow-mediated dilation (FMD), and the levels of soluble adhesion molecules, are significantly correlated with the levels of atherosclerosis markers (123). Recent studies have shown that quercetin (QC) reduces the number of senescent follicular helper T (Tfh) cells, inhibits Tfh cell differentiation, and increases the apoptosis of senescent CD4+ T cells (124). This may help to improve lupus symptoms and slow atherosclerosis.

Natural killer T (NKT) cells bridge innate and adaptive immunity. They are activated in both SLE and atherosclerosis (AS) and can rapidly produce large amounts of proinflammatory or anti-inflammatory cytokines, such as IFN-γ and IL-4. Impaired function of an NKT cell subset, invariant NKT cells (iNKT), has been reported in SLE and appears to be associated with autoimmunity and atherosclerosis (125).

### 4.2 B cells: a dual role in atherogenesis

B cells are central to the pathology of SLE, but the role of these cells and their antibodies in atherosclerosis remains controversial, with B cells exerting both protective and pathogenic effects. B cells were once thought to be sparse in plaques and not play a major role. Subsequently, these cells were considered anti-atherosclerotic immune cells. B-cell-deficient LDLR^-/-^ mice exhibited increased atherosclerosis after 4 weeks of high-fat diet feeding. Their serum cholesterol levels were not significantly different from those of LDLR^-/-^ mice, with WT mice serving as bone marrow donors, but total serum antibodies and antibodies against ox-LDL were significantly reduced (126), indicating that the absence of natural B cells accelerates the AS process.

However, in a long-term autoimmune environment, B cells can instead promote atherosclerosis. For example, researchers found that using monoclonal antibodies targeting CD20+ to deplete mature B cells significantly reduced atherosclerosis in various disease mouse models (127). This treatment preserved the production of natural and potentially protective anti-oxLDL IgM autoantibodies while significantly reducing the production of IgG antibodies, decreasing T-cell-derived IFN-γ secretion, and increasing IL-17 production. As research has progressed, B cells can be divided into B1 and B2 cells according to their developmental pathways, with B1 cells having anti-atherosclerotic effects and B2 cells promoting atherosclerosis (128). More recent studies have provided a more detailed classification of the roles of B-cell subsets in atherosclerosis. Marginal zone B (MZB) cells exert protective effects by regulating Tfh cells and possibly through the secretion of IgM, whereas follicular B (FOB) cells are considered to have pro-atherosclerotic effects because they can undergo GC reactions and form class-switched plasma cells (129). T-cell-dependent B-cell responses can also occur in adventitial tertiary lymphoid organs (ATLOs), which form around late-stage atherosclerotic lesions and are proposed to have anti-atherosclerotic effects (130).

Belimumab is the first biologic agent approved for the treatment of systemic lupus erythematosus (SLE), raising the question of whether BAFF inhibition can also prevent atherosclerosis in patients with this disease. The relationship between BAFF inhibition and atherosclerosis prevention in SLE is complex and context-dependent (131). While high circulating levels of BAFF are consistently linked to increased subclinical vascular disease among SLE patients, interventional studies reveal that blocking BAFF can have both beneficial or detrimental effects depending on metabolic status, immune cell context, and possibly genetic background.

Animal models show that while anti-BAFF therapy reduces lupus activity and can decrease plaque burden under certain conditions (low cholesterol), it paradoxically worsens lesions when hyperlipidemia is present—likely due to loss of anti-inflammatory signaling via TACI receptors on myeloid cells. This effect underscores why simple extrapolation from immunosuppression to cardiovascular benefit is not always valid (132). This may be because the atherosclerosis-protective effect of B-cell-mediated BAFF–BAFF receptor signaling inhibition is offset by the pro-atherosclerotic effect of BAFF–TACI signaling inhibition, which promotes macrophage transformation into foam cells. Moreover, the atherosclerosis-protective effects mediated by BAFF overexpression are also associated with a significant increase in TACI-dependent natural IgM antibody titers (133). However, recent studies have shown that Belimumab treatment may also increase the atherosclerosis-protective effects of HDL in patients with SLE and alter the lipidomic profile of HDL, thereby reducing atherosclerosis (134). The role of BAFF in atherosclerosis remains controversial, indicating that clinicians should closely monitor cardiovascular conditions in patients treated with BAFF inhibitors for lupus. Given these findings and the fact that some forms of global B cell depletion do not reduce CVD risk, future research should focus on precision targeting within immune pathways rather than broad suppression alone.

### 4.3 Antibodies: protective versus pathogenic roles

Autoantibodies targeting different types of antigens, including ox-LDL and its epitopes (such as MDA and PC), vascular wall antigens (such as HSP60 and GRP78), mitochondrial antigens (such as ALDH4A1), and classical autoantigens (such as β2GPI and cardiolipin), have been proposed to be associated with atherosclerotic cardiovascular disease (ACVD) (129). Different types of antibody responses exist against these antigens, and protective and pathogenic effects are produced depending on the antigen and antibody isotypes (93).

Preexisting natural IgM antibodies can prevent the development of atherosclerosis, whereas IgG autoantibodies that undergo class-switching against relevant oxidized lipid epitopes promote atherosclerosis development. In patients with SLE, a reduction in protective antibodies and an increase in pathogenic antibodies together lead to atherosclerosis. In a cohort study of 434 SLE patients and 322 age-matched controls, compared with those without cardiovascular disease, SLE patients with cardiovascular disease had lower levels of p210 IgG and p45 IgM (135). The increased incidence of carotid plaques in SLE patients is associated with low levels of anti-PC IgM (136). In SLE patients with high triglyceride levels or low HDL levels, increased CD4+CD28 null and Th17 cells, along with reduced IgM anti-PC and Tregs, may reveal the protective mechanism of anti-PC IgM against atherosclerosis (137). Analysis of antiphospholipid antibodies in 93 patients with SLE and 30 controls revealed that patients with antibodies against phosphatidylethanolamine (aPE) and phosphatidylserine (aPS) had a higher risk of vascular involvement.

IgG is the most abundant antibody isotype in the body and accounts for more than half of the total serum antibodies. Large amounts of IgG molecules are deposited at the sites of organ and tissue damage in lupus mice, and injecting serum from lupus mice into healthy mice induces inflammation in tissues and organs, which does not occur with serum from other healthy mice (138). It is now believed that most IgG is pro-atherosclerotic. However, its effects on atherosclerosis vary depending on the specific antigen. IgG responses against β2GP1, HSP60, GRP78, and cardiolipin lead to increased plaque development in mice, whereas IgG responses against MDA-OxLDL, PC, and pCETP show anti-atherosclerotic effects (139).

IgE has the same role in the pathogenesis of SLE. Autoreactive IgE increases basophil activation, promotes the accumulation of DNA-containing immune complexes in phagosomes and increases IFN-α production. Non-autoreactive IgE may act as a negative regulator of IFN-α production and inhibit lupus (140). For example, in SLE, IgE anti-dsDNA and anti-SSA/Ro52 antibodies are associated with disease activity and skin manifestations (141). IgE and FcϵR1 are highly expressed in unstable atherosclerotic plaques in humans and mice (142). In the inflammatory pathway of psoriasis-exacerbating atherosclerosis, high IgE production may originate from IL-17A (143), and a similar pathway may be involved in SLE.

Among autoantibodies in SLE, antiphospholipid antibodies (aPL) are a major, independent cardiovascular risk factor (144). aPL are present in roughly one−third of SLE patients and are strongly associated with venous and arterial thrombosis, cerebrovascular events and future atherosclerotic cardiovascular disease, as well as non−thrombotic cardiac manifestations (145, 146). When persistent aPL positivity coexists with thrombosis or pregnancy morbidity, patients meet criteria for antiphospholipid syndrome (APS), in whom cardiovascular events are a leading cause of morbidity and mortality (147). These patients require distinct management, including risk−stratified antithrombotic prophylaxis: Low−dose aspirin for high−risk aPL−positive SLE without prior events, and long−term vitamin K antagonist anticoagulation after thrombotic events, rather than standard cardiovascular prevention alone (148, 149).

## 5 Treatment strategies for systemic lupus erythematosus atherosclerosis and exploration of novel immunotherapies

### 5.1 Pharmacological treatments and atherosclerosis in SLE

Major position statements converge on a strategy that combines intensive control of traditional risk factors with SLE−specific measures (150). Carotid ultrasound independently predicts cardiovascular events and substantially improves risk reclassification (151). Adding carotid imaging can triple correct identification of high−risk SLE patients (152). Regular vascular imaging is not yet universally mandated, but many centers use carotid ultrasound in SLE patients with intermediate risk or additional disease−related risk factors.

Prolonged glucocorticoid exposure is consistently linked to heightened atherosclerotic and cardiovascular risk in SLE (152). Chronic use, especially higher daily doses, is associated with increased CVD events, likely via worsening lipids, blood pressure, body weight, and possibly direct pro−atherogenic plaque effects (153). Contemporary recommendations therefore stress strict steroid minimization as a core cardiovascular prevention strategy (22, 154).

Hydroxychloroquine (HCQ) is the only disease−modifying therapy with reproducible cardioprotective signals in SLE (155). HCQ’s benefits appear to require continuous, long−term use (156). Remote or intermittent exposure may not confer persistent protection. Current EULAR cardiovascular recommendations explicitly endorse HCQ for all SLE patients without contraindications, partly for its potential to reduce CV events (22).

Non−aspirin NSAIDs increase cardiovascular risk in the general population, with higher rates of thrombotic events, heart failure, and blood pressure elevation, particularly for COX−2–selective agents and diclofenac (157). Given SLE patients’ already elevated baseline risk, long−term or high−dose NSAID use should be approached cautiously, favoring the lowest effective dose and shortest possible duration, and avoiding them in patients with established CVD when alternatives exist.

### 5.2 Exploring immunotherapy approaches for SLE-associated accelerated atherosclerosis

Current clinical practice still recommends controlling cardiovascular risk factors and lupus disease activity as the primary measures for reducing SLE-related vascular disease (93, 158). However, targeting the complex immunopathological mechanisms in autoimmune diseases, multiple therapeutic approaches directed at the immune system are currently being explored. At the cytokine level, the IFN-I pathway represents a key target. Treatment of SLE patients with JAK inhibitors reduces expression of type I interferon-related genes, improves lipoprotein function, and demonstrates potential cardiovascular protective effects (159). Monoclonal antibodies targeting IFN-α have been approved for SLE treatment, and their impact on atherosclerotic progression warrants further investigation (160, 161). Therapies targeting other proinflammatory cytokines also show promise. A novel bispecific antibody targeting TNF-α and IL-6 receptors has recently been discovered (162). Regarding regulatory T cell responses, mycophenolate mofetil slows disease progression by inhibiting CD4+ T cell activation and infiltration into atherosclerotic lesions (163, 164). Additionally, therapeutic strategies targeting the neutrophil/NETosis pathway (e.g., targeting BTK, tRF-His-GTG-1), macrophage cholesterol efflux (e.g. artemisinin, resveratrol), and the complement system (e.g., targeting C5a, membrane attack complex) are emerging as potential therapeutic avenues for intervening in SLE-associated atherosclerosis (111, 165–168). In the future, selecting personalized combinations of targeted immunotherapies based on precise patient immune profiling may be key to achieving effective prevention and treatment of SLE atherosclerosis.

## 6 Conclusions and future directions

Systemic lupus erythematosus (SLE), a systemic autoimmune disease, has complex and multilayered mechanisms underlying its associated atherosclerosis (AS), involving extensive dysregulation of both the innate and adaptive immune systems. These mechanisms collectively form a complex immune–inflammatory network, leading to endothelial dysfunction, lipid metabolism disorders, plaque formation and instability, ultimately accelerating the AS process. Although numerous studies have revealed the close link between SLE and AS, many mysteries remain to be explored through further research. Although improved risk scoring models incorporating parameters such as disease activity scores, inflammatory cytokines, or autoantibodies have been proposed, their application has not yet been widely adopted, and their impact on cardiovascular outcomes still needs to be verified. Therefore, it is necessary to assess and monitor arteriosclerotic cardiovascular disease (ASCVD)in these patients as early as possible through imaging and laboratory biomarkers. Future research can focus on several aspects. First, emerging technologies can be used to further explore the mechanisms of SLE autoimmune-mediated accelerated atherosclerosis. Second, targeted therapeutic strategies for specific immune pathways can be developed. Third, a cardiovascular risk assessment system specific to SLE patients can be established to achieve early identification and intervention and to promote the development of personalized treatment. Emerging data indicate that gut dysbiosis and microbiota−derived metabolites, including pro−atherogenic compounds, can modulate systemic immune responses, endothelial function, and lipid metabolism, and may therefore contribute to SLE−associated atherosclerosis (169, 170). Future studies integrating microbiome, metabolomic and immunologic profiling in SLE are needed to clarify these pathways and therapeutic opportunities (171, 172).

In summary, the promotion of atherosclerosis by SLE is a complex process involving multiple cells and pathways. A deeper understanding of its mechanisms not only helps to reveal the intrinsic connections between autoimmunity and cardiovascular disease but also provides an important scientific basis for the development of new therapeutic strategies and the improvement of patient prognosis.
